# Interfractional robustness of scanning carbon ion radiotherapy for prostate cancer: An analysis based on dose distribution from daily in‐room CT images

**DOI:** 10.1002/acm2.13275

**Published:** 2021-05-27

**Authors:** Keisuke Tsuchida, Shinichi Minohara, Yohsuke Kusano, Kio Kano, Wataru Anno, Yosuke Takakusagi, Nobutaka Mizoguchi, Itsuko Serizawa, Daisaku Yoshida, Koh Imura, Yoshiki Takayama, Tadashi Kamada, Hiroyuki Katoh, Tatsuya Ohno

**Affiliations:** ^1^ Department of Radiation Oncology Kanagawa Cancer Center Yokohama Kanagawa Japan; ^2^ Section of Medical Physics and Engineering Kanagawa Cancer Center Yokohama Kanagawa Japan; ^3^ Department of Radiation Oncology Gunma University Graduate School of Medicine Maebashi Gunma Japan

**Keywords:** carbon ion radiotherapy, image‐guided radiotherapy, in‐room CT, interfractional movement, prostate cancer, scanning irradiation

## Abstract

**Purpose:**

We analyzed interfractional robustness of scanning carbon ion radiotherapy (CIRT) for prostate cancer based on the dose distribution using daily in‐room computed tomography (CT) images.

**Materials and Methods:**

We analyzed 11 consecutive patients treated with scanning CIRT for localized prostate cancer in our hospital between December 2015 and January 2016. In‐room CT images were taken under treatment conditions in every treatment session. The dose distribution on each in‐room CT image was recalculated, while retaining the pencil beam arrangement of the initial treatment plan. Then, the dose–volume histogram (DVH) parameters including the percentage of the clinical target volume (CTV) with 95% and 90% of the prescribed dose area (V95% of CTV, V90% of CTV) and V80% of rectum were calculated. The acceptance criteria for the CTV and rectum were set at V95% of CTV ≥95%, V90% of CTV ≥98%, and V80% of rectum < 10 ml.

**Results:**

V95% of CTV, V90% of CTV, and V80% of rectum for the reproduced plans were 98.8 ± 3.49%, 99.5 ± 2.15%, and 4.39 ± 3.96 ml, respectively. Acceptance of V95% of CTV, V90% of CTV, and V80% of rectum was obtained in 123 (94%), 125 (95%) and 117 sessions (89%), respectively. Acceptance of the mean dose of V95% of CTV, V90% of CTV, and V80% of rectum for each patient was obtained in 10 (91%), 10 (91%), and 11 patients (100%), respectively.

**Conclusions:**

We demonstrated acceptable interfractional robustness based on the dose distribution in scanning CIRT for prostate cancer.

## 1 INTRODUCTION

Prostate cancer is the second most prevalent and fifth most lethal among all cancers according to a global cancer database.^1^ Both radiotherapy (RT) and surgery produce similar clinical outcomes and play important roles as radical treatments for localized prostate cancer.^2^ RT Technological improvements have enabled physicians to provide patients with various treatment modalities, including intensity‐modulated RT (IMRT), brachytherapy, and particle therapy.^2^ Among particle therapies, the effectiveness of both proton and carbon ion RT (CIRT) was demonstrated by previous studies.^3^, ^4^, ^5^, ^6^, ^7^, ^8^, ^9^, ^10^, ^11^, ^12^

Cancer treatment using CIRT was introduced at the National Institute of Radiological Sciences in Chiba, Japan in 1994, following pioneering research for heavy‐ion radiotherapy at Lawrence Berkeley National Laboratory. Already in 1995, the first clinical trial of CIRT was started for prostate cancer.^5^ Compared with conventional RT with x‐rays, CIRT has clear physical and biological advantages, such as an approximately threefold greater relative biological effectiveness (RBE) of carbon ion beams.^13^, ^14^ From physical perspective, an improved dose distribution of the carbon ion beam stems from releasing the maximum amount of energy at the end of the accelerated carbon ions track, which results in a Bragg peak.^15^ Hence, dose escalation can be achieved for tumor tissues with notably lower toxic effects on normal tissues.

However, dose distribution of particle therapy displays particular sensitivity to variations in the internal density along the beam pathway from the skin surface to the target’s distal edge; this issue is caused by anatomical factors including volume of gas in the rectum and urine in the bladder. Therefore, the high reproducibility of patient positioning including the situation around the internal target is essential for ensuring an accurate dose distribution during actual treatment sessions in particle therapy.^16^, ^17^

Particle therapy facilities have developed image‐guided RT (IGRT) methods.^18^ Cone beam computed tomography (CBCT) is often applied in photon RT, and its effectiveness was demonstrated.^19^ Nevertheless, the accuracy of computed tomography (CT) numbers for CBCT images is not sufficient to calculate the dose distribution of particle therapy compared with that of fan‐beam CT images.^18^ In‐room fan‐beam CT (in‐room CT) has the same quality as the CT performed during the initial treatment planning, and it maintains the same patient position on the couch during CT image acquisition and irradiation.

Several previous studies evaluated the robustness of particle therapy using in‐room CT. In proton therapy for prostate cancer, prostate movements and the reproducibility of dose distributions in actual treatment sessions based on daily and weekly in‐room CT images were analyzed.^20^, ^21^, ^22^ In CIRT, interfractional changes in the anatomy and dose distribution of passive CIRT were reported using daily in‐room CT.^23^ However, the dose distribution of the pencil beam scanning method is more sensitive to anatomical factors than that of the passive beam control method.^24^ Although the reproducibility of the dose distribution of scanning CIRT was evaluated in a previous study, the study did not apply the daily in‐room CT for the evaluation.^25^

Our facility started the clinical operation of CIRT using the raster scanning method with fixed vertical and horizontal beam ports in 2015. Each treatment room has an in‐room CT image‐guided system in addition to an orthogonal x‐ray flat‐panel detector (FPD) imaging system for the positioning the patient based on bone structures. Of note, the geometrical arrangement of the in‐room CT system is the same as in the CT simulation room for treatment planning. Here, we analyzed the interfractional robustness of dose distribution using a complete series of daily in‐room CT images in scanning CIRT for prostate cancer.

## 2 MATERIALS AND METHODS

### 2.A Patients

We analyzed 11 consecutive patients treated with CIRT for localized prostate cancer in our hospital in the period from December 2015 to January 2016. We defined the following inclusion criteria: (i) prostate adenocarcinoma (histologically confirmed), (ii) stage cT1bN0M0 to T3bN0M0 according to the seventh UICC classification, (iii) aged ≥20 yr, (iv) ECOG performance status of 0–2, and (v) lack of previous treatment received for prostate cancer except androgen deprivation therapy. We classified the patients based on the D’Amico risk group classification.^26^ All patients gave written informed consent and the institutional review board in our hospital (approval number: 27–40) approved this study.

### 2.B Immobilization and data acquisition

Each patient was placed on a vacuum mattress (BlueBAG: Elekta AB, Stockholm, Sweden) in the supine position and immobilized with thermoplastic shells (Shellfitter: Kuraray, Tokyo, Japan). Laxatives and antiflatulents were used before planning CT and each treatment session to empty the rectum as much as possible. Enema was routinely used before planning CT. In each treatment session, enema was used only for patients who did not defecate for 24 hr before treatment. For bladder filling, each patient urinated and drank 250 ml of water 40 min prior to entering the planning CT and treatment room. For treatment planning, we took a set of 2‐mm thick CT slices.

**Fig. 1:**
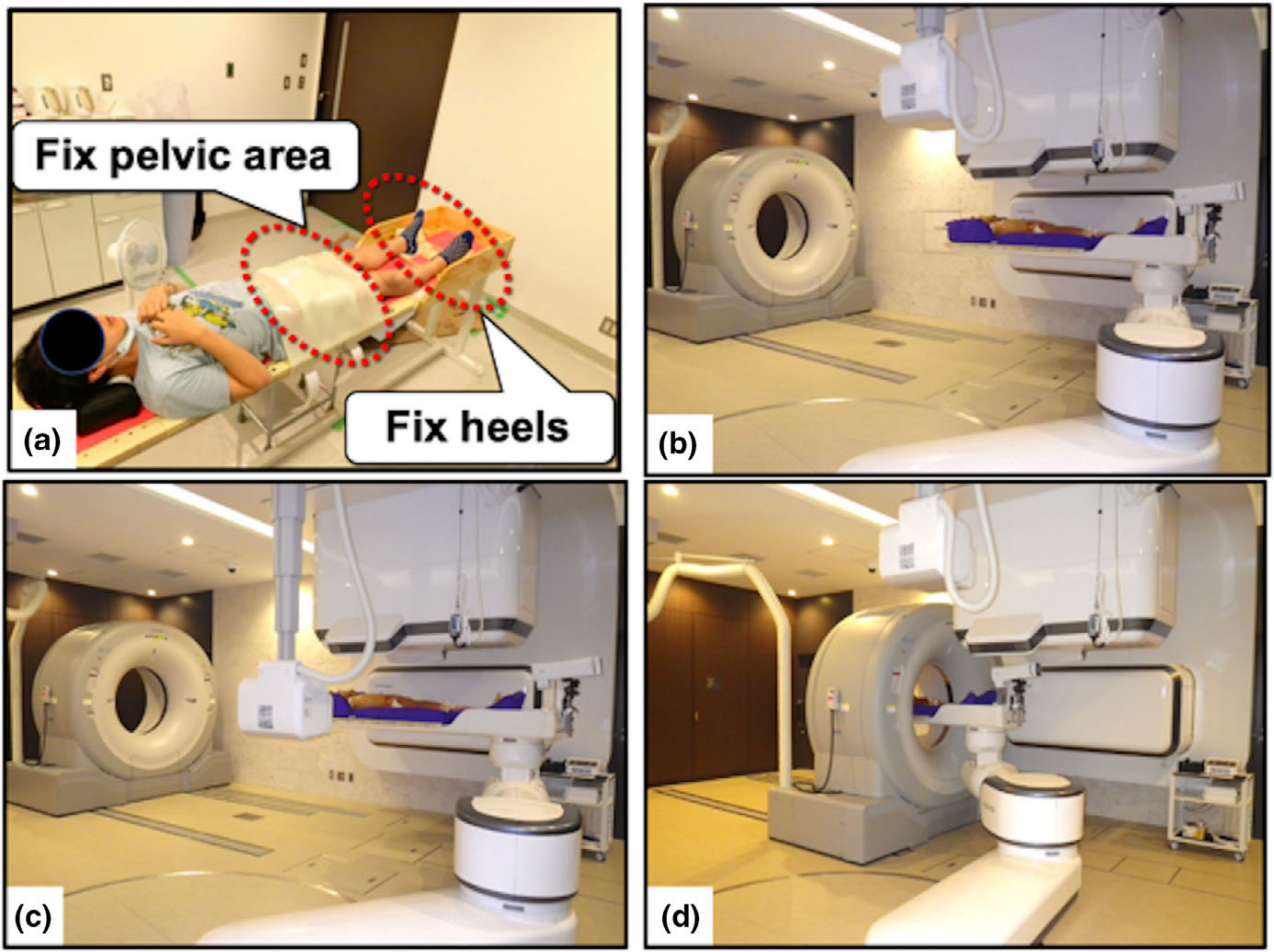
(a) Immobilization of a patient with prostate cancer. The patient moved to the treatment position (b), and we acquired a two‐dimensional (2D) orthogonal x‐ray image and compared bone structure between the X‐ray and planning computed tomography (CT) images. The patient was moved to the correct position automatically (c). Then, we acquired in‐room CT images and verified that patient set‐up was acceptable (d).

A computer‐aided online 2D–3D positioning program using orthogonal kilovoltage (kV) x‐ray FPD images was initially employed in each treatment session to position the patient with an accuracy of <1 mm based on bone structures. Immediately before or after irradiation during this study period, in‐room CT images were taken under the treatment conditions in every treatment session. Patient set‐up was performed using kV x‐ray FPD images only based on bony anatomy. The actual details of set‐up and treatment are presented in Fig. 1.

### 2.C Treatment planning

MIM maestro software version 5.6. (MIM Software Inc., Cleveland, OH, USA) was used to contour the target volumes as well as normal tissues. Monaco version 5.20 for carbon scanning system (Elekta AB, Stockholm, Sweden) was used to calculate and optimize the dose. The system has a smearing parameter which is a robust optimization algorithm.

We did not define the gross tumor volume. The entire prostate and proximal seminal vesicles were included in the clinical target volume (CTV). The ipsilateral seminal vesicles were included in the CTV in case of T3b stage of prostate cancer. We set the planning target volume (PTV) as described in previous CIRT studies.^6^, ^9^ To create PTV1, the CTV was expanded by 10mm on the superior, inferior, anterior and lateral margins and 5 mm on the posterior margin. For boost therapy using PTV2, from the ninth course of treatment, the posterior margin was marked in front of the anterior rectum’s wall to achieve a lower rectal dose. The delineation of the rectum as the organ at risk stretched from 10 mm superior to the PTV’s upper margin to 10 mm inferior to its lower margin.

The total dose of 51.6 Gy (RBE) was divided into 12 fractions. PTV1 was used for the first eight fractions, whereas PTV2 was used for boost therapy. The PTV was covered by at least 95% of the prescribed dose, and we ensured that the maximum PTV dose did not exceed 105% of the prescribed dose. We aimed at V80% <10 ml for the rectum. Two horizontally opposing irradiation fields were delivered to each patient, and radiation was delivered to one field per day on 4 days a week over a 3‐week period.

### 2.D Data analysis

After the acquisition of in‐room CT images, the treatment plan based on the initial planning CT images was reconstructed using in‐room CT images and MIM Maestro. Namely, rigid image fusion was performed via bony structure matching between planning CT and in‐room CT, and the structures (CTV, bladder, and rectum) were reproduced on in‐room CT images by two experienced radiation oncologists. Then, we calculated simulated dose distribution was recalculated on in‐room CT images. The dose–volume histogram (DVH)‐derived parameters, including the percentage of CTV that included 95% of the prescribed dose area (V95% of CTV), V90% of CTV and V80% of rectum were calculated. The acceptance criteria of the CTV and rectum were set at V95% of CTV ≥95%, V90% of CTV ≥98%, and V80% of rectum <10 ml. The movement of the CTV center and correlation between the DVH and volume of the rectum and bladder were also analyzed.

### 2.E Statistical analysis

We used IBM SPSS Statistics software, version 26.0 (IBM, Armonk, NY, USA) for all statistical analyses. We determined the strength of associations among the bladder volume, rectal volume, movement of the CTV, and dose coverage of the CTV and rectum using Pearson’s correlation coefficient.

## 3 RESULTS

### 3.A DVH parameters and dose distribution

The characteristics of patients along with clinical results are summarized in Table 1. We obtained data for 11 patients with prostate cancer, and 131 in‐room CT datasets were available (one dataset was missed). The median follow‐up period for all patients was 54 (range 47–56) months.

**Table 1:** Patients characteristics.

Patient no.	Age	T factor	GS	initial PSA	D'Amico risk	Survival	Recurrence	Acute AE	Late AE
1	73	1c	4 + 3	10.8	intermediate	yes	no	none	none
2	71	3a	4 + 4	23.4	high	yes	no	Urinary frequency G1	none
3	73	1c	3 + 4	6.1	intermediate	yes	no	none	none
4	71	3a	3 + 4	13.5	high	yes	no	Urinary retention G1	Urinary incontinence G2
5	71	1c	3 + 4	5.0	intermediate	yes	no	none	none
6	74	3a	4 + 3	4.9	high	yes	no	none	none
7	78	3a	3 + 3	7.9	high	yes	no	none	none
8	71	3a	4 + 5	25.9	high	yes	no	Urinary retention G1	Urinary retention G1
9	78	1c	4 + 3	5.9	intermediate	yes	no	Urinary frequency G2	none
10	66	3b	4 + 5	148.0	high	yes	no	none	none
11	67	3a	4 + 5	84.0	high	yes	no	none	none

Abbreviations: AE, Adverse event; GS, Gleason score; PSA, Prostate‐specific antigen.

**Fig. 2:**
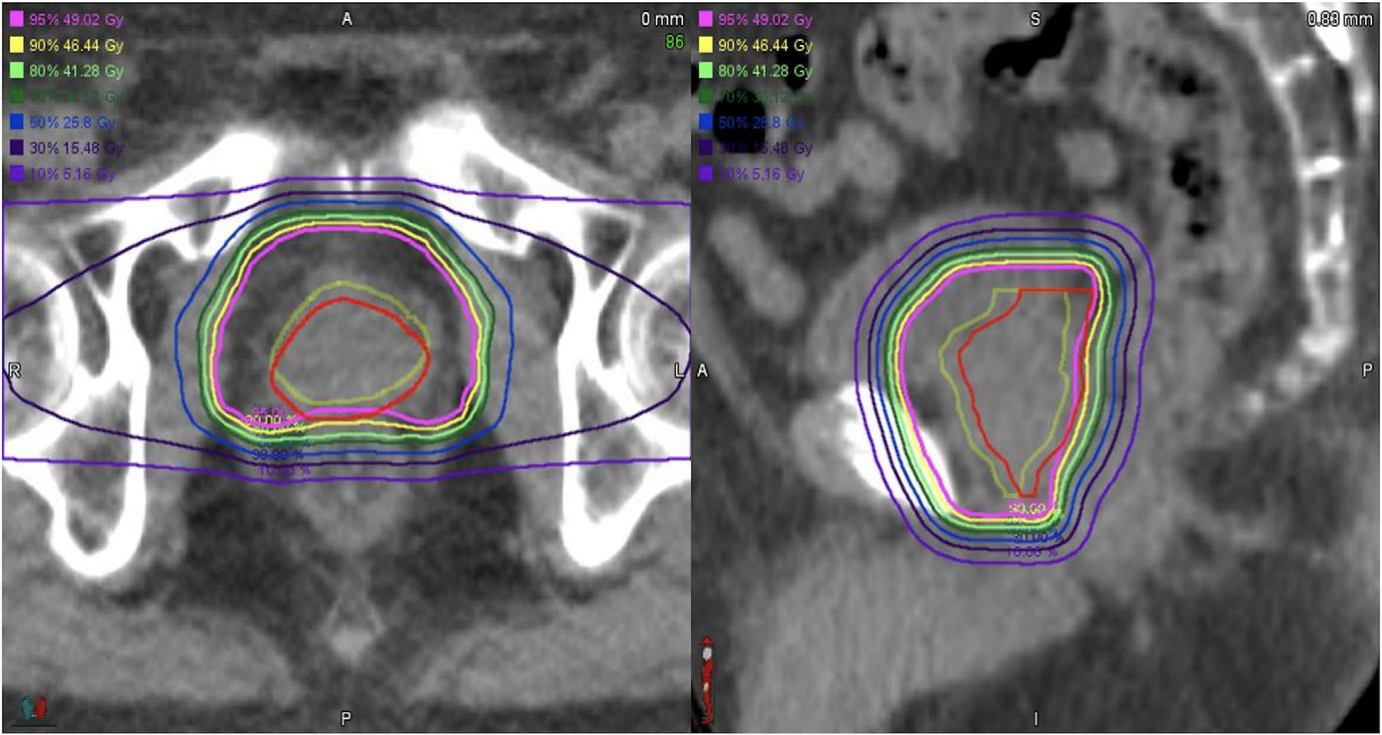
Representative reproduction of the dose distribution. The yellow contoured line shows the clinical target volume (CTV) on planning computed tomography (CT), and the red line denotes the CTV on in‐room CT.

Figure 2 presents a representative example of the dose distribution reproduced on in‐room CT. We observed the translocations of the prostate and rectum between planning and in‐room CT and the differences of the dose to such organs visually.

**Fig. 3:**
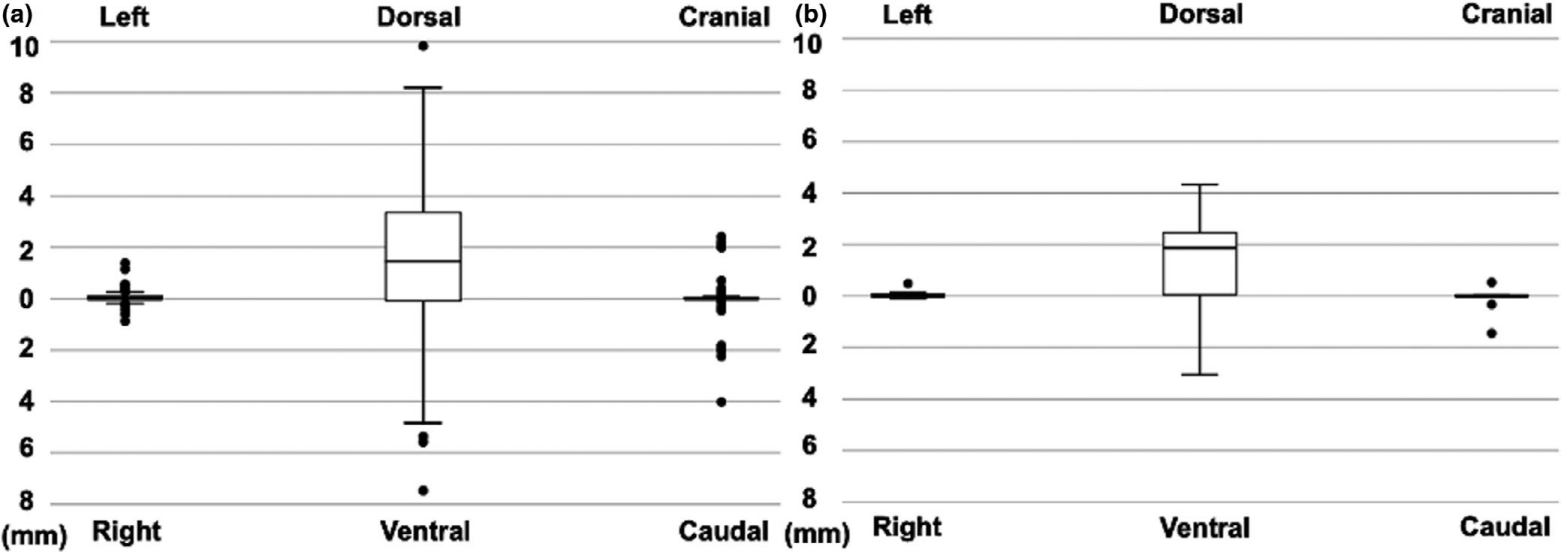
Box‐whisker plot of the movement of the center of the clinical target volume (CTV) from planning computed tomography (CT) to in‐room CT. (a) Plots for each session. (b) Mean plots for each patient.

The movement of the CTV center in each direction is presented in Fig. 3. The CTV center scarcely moved to the lateral and craniocaudal direction (mean ± standard deviation were 0.05 ± 0.24 mm for the lateral direction and −0.10 ± 0.90 mm for the craniocaudal direction). However, the CTV center tended to move posteriorly (mean ± standard deviation was −1.34 ± 2.83 mm for the ventrodorsal direction) in some sessions.

**Fig. 4:**
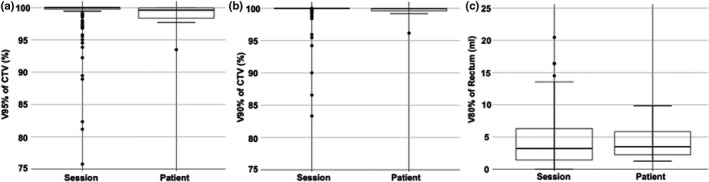
Box‐whisker plots of the dose–volume histogram (DVH) parameters of the clinical target volume (CTV) and rectum. Left plots present the DVH parameters for each session, and the right panels display the mean parameters of each patient.

Figure 4 presents the DVH parameters of the initial treatment plan for each patient and that of the recalculated dose distribution on in‐room CT images (retain plan), while retaining the pencil beam arrangement of the initial plan. V95% of CTV, V90% of CTV, and V80% of rectum for all sessions were 98.8 ± 3.49%, 99.5 ± 2.15%, and 4.39 ± 3.96 ml, respectively. Acceptance of V95% of CTV, V90% of CTV, and V80% of rectum was achieved in 123 (94%), 125 (95%), and 117 sessions (89%), respectively. In the cases in which V95% or V90% of CTV were unacceptable, the lesion for which dose degradation occurred was located on the posterior side of the CTV in every case. Concerning each patient, the mean doses of all sessions were analyzed. Acceptance of the mean dose of V95% of CTV, V90% of CTV, and V80% of rectum for each patient was obtained in 10 (91%), 10 (91%), and 11 patients (100%), respectively.

### 3.B Assessment of factors correlated with dose coverage

**Fig. 5:**
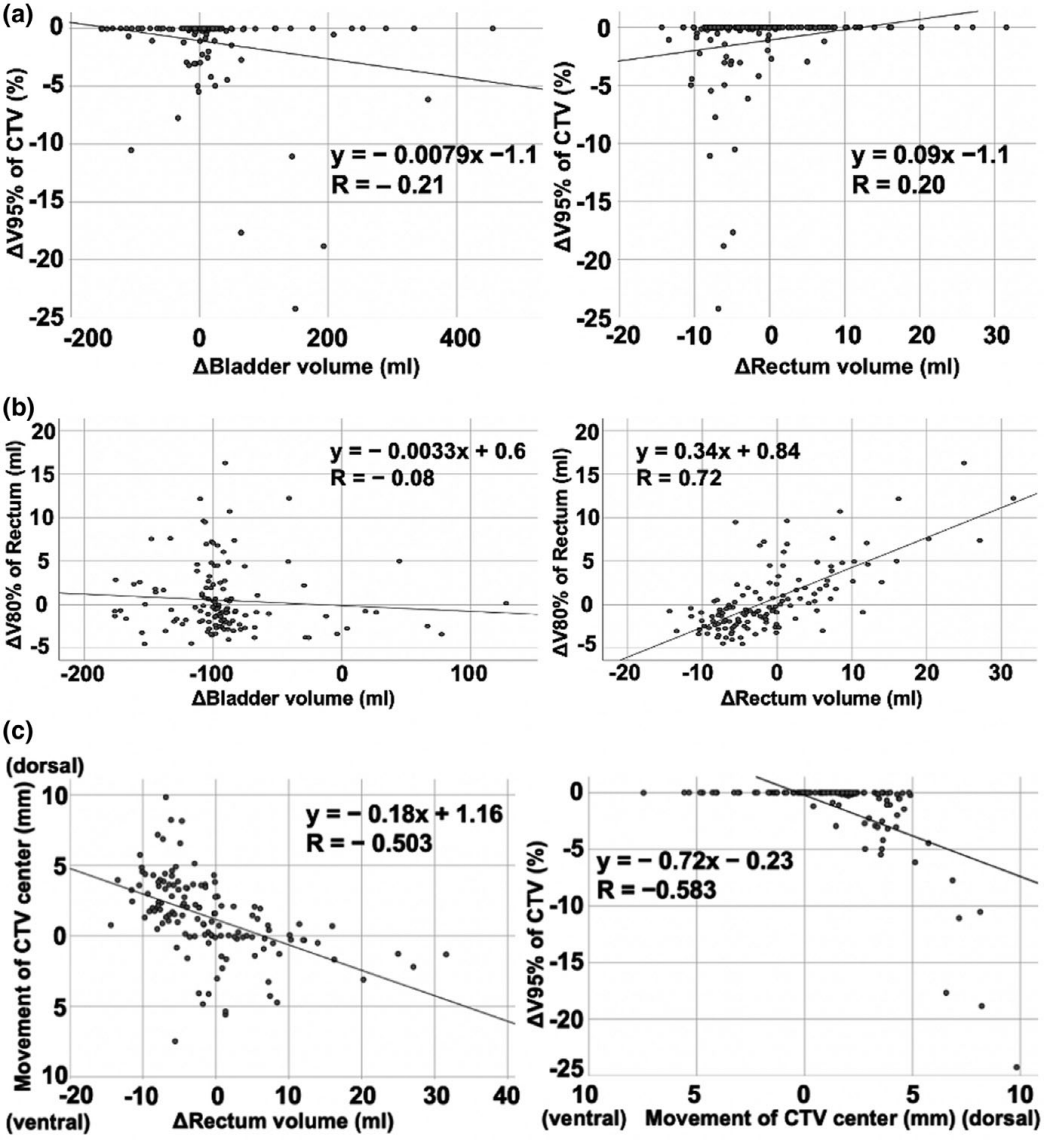
(a) Scatter plot of the correlations of ΔV95% of the clinical target volume (CTV) with Δbladder volume and Δrectal volume for each session. The lines express the linear approximate lines. (b) Scatter plot of the correlations of ΔV80% of rectum with Δbladder volume and Δrectal volume for each session. The lines express the linear approximate lines. (c) Scatter plot of the correlations of the movement of the CTV center, Δrectal volume, and ΔV95% of CTV. The lines express the linear approximate lines.

The correlations between DVH and variations of the bladder and rectal volume are presented in Fig. 5. ΔBladder volume determined as the difference between bladder volume in in‐room CT and bladder volume in planning CT, and Δrectal volume ([rectal volume in in‐room CT] − [rectal volume in planning CT]) were 151.5 ± 95.2 and −1.00 ± 7.91 ml, respectively. The correlation coefficients of ΔV95% of CTV ([V95% of CTV in in‐room CT] − [V95% of CTV in planning CT]) in each session with Δbladder volume and Δrectal volume were −0.21 and 0.20, respectively. The CTV dose was not unacceptable (V95% of CTV was <95%) in any sessions in which Δrectal volume was ≥0 ml. Moreover, the CTV dose was unacceptable in eight sessions (9.2%) in which Δrectal volume was <0 ml (*P* = 0.034, Fisher’s exact test), and these eight sessions were in three patients.

Regarding the correlations of the movement of the CTV center with Δrectal volume and ΔV95% of CTV, the correlation coefficient between Δrectal volume and ventrodorsal movement of the CTV center was −0.503. The correlation coefficient between the ventrodorsal movement of the CTV center and ΔV95% of CTV was −0.583.

The correlation coefficients of Δbladder volume and Δrectal volume with ΔV80% of rectum for each session ([V80% of rectum in in‐room CT] − [V80% of rectum in planning CT]) were −0.08 and 0.72, respectively.

## 4 DISCUSSION

This study aimed to verify the interfractional robustness of scanning CIRT for prostate cancer based on the dose distribution using a complete series of daily in‐room CT image datasets. Prior studies analyzed the interfractional robustness of IMRT^16^, ^17^, ^22^, ^27^, ^28^ and proton therapy^16^, ^17^, ^20^, ^21^, ^22^, ^29^, ^30^, ^31^ using various types of CT images. In CIRT, the robustness of the dose distribution of passive CIRT was analyzed using daily in‐room CT datasets.^23^ Moreover, the robustness of dose distributions of scanning CIRT for prostate cancer with or without spacer gel was analyzed by a prior study, in which daily in‐room CT was not applied.^25^ Therefore, this study is the first to analyze the interfractional robustness of scanning CIRT for prostate cancer based on the dose distribution using complete series of daily in‐room fan‐beam CT image datasets in the actual treatment setting.

We analyzed CTV movement using the CTV center as a representative point. Zhang et al. reported the largest movement of the prostate in the ventrodorsal direction when comparing skin marker matching and prostate matching methods. The average movement in the ventrodorsal direction exceeded 5 mm in 3 of 11 patients.^17^ Seo et al. reported that interfractional prostate movements after bone matching were more profound in the ventrodorsal and craniocaudal directions (2.14 ± 1.73 and 1.97 ± 1.44 mm, respectively) than in the lateral direction (0.26 ± 0.22 mm). Prostate movements were analyzed using fiducial markers.^32^ In this study, the patient was placed in isocenter via a bone‐matching method using orthogonal x‐ray FPD images, and then the in‐room CT images were taken. CTV movements were analyzed using in‐room CT images. The CTV center scarcely moved in the lateral and craniocaudal directions. However, the CTV center tended to move dorsally in some sessions, degrading dose coverage of the CTV. In seven of eight sessions in which V95% of CTV is degraded beyond the acceptable limit, the movement of the CTV center in the dorsal direction was 5 mm or more. Therefore, although we must consider the trade‐off relationship between the CTV and rectal dose, we should pay attention to CTV movement, particularly in the posterior direction, to ensure that CTV coverage is not degraded.

Previous studies examined robustness based on the dose distribution for IMRT, proton therapy, and CIRT with different alignment methods, as summarized in Table 2. Zhang et al. used in‐room CT and analyzed the robustness of proton therapy and IMRT. They reported that CTV coverage was better for the prostate center matching method than for the skin marker matching method, whereas the rectal dose was better for the skin marker matching method.^17^ Maeda et al. compared robustness between the prostate and bone matching methods for proton therapy in prostate cancer patients using in‐room CT. They demonstrated that the prostate matching method was superior to the bone matching method regarding both target coverage and rectal sparing.^20^ Wang et al. also reported acceptable robustness for proton therapy using the prostate matching method.^29^ Concerning CIRT, Rucinski et al. reported the robustness of CIRT for prostate cancer with and without spacer gel. They used weekly in‐room CT for no‐spacer data and 1–2 times CT in the other room for spacer‐data to reproduce the dose distribution. In addition, they applied the femur bone matching method. They demonstrated the utility of spacer gel, especially for the rectal dose.^25^ Yokoyama et al. reported the robustness of dose distribution of passive CIRT using daily in‐room CT images. Good reproducibility is verified in both target (prostate) and rectum dose applying the bone matching method.^23^

**Table 2:** Interfractional dose assessments of previous studies and this study.

Author	Treatment	Prescribed dose	Number of datasets	Alignment method	Modality for evaluation	Target coverage			OAR dose		
						Parameter	Plan (Mean ± SD )	Reproduction (Mean ± SD )	Parameter	Plan (Mean ± SD )	Reproduction (Mean ± SD )
Zhang et al. ^17^	IMRT	75.6 Gy/42fr.	80 (10 patients)	Skin‐marker matching	CT‐on‐rail	V100% of CTV	96.20%	94.50%	V60 Gy of rectum	27.20%	29.40%
				Prostate center matching			100%	95.60%		21.50%	28.80%
	Proton			Skin‐marker matching			100%	96.30%		32.60%	33.60%
				Prostate center matching			100%	98.20%		23.70%	30.80%
Wang et al.^29^	Proton	78 Gy/39 fr.	33 (Patient 1)	Prostate center matching	CT‐on‐rail	V100% of PTV2	98.60%	98.50%	V70 Gy of rectum (Reproduction ‐ Plan)	N/R	‐0.30%
			36 (Patient 2)				98.10%	96.60%		N/R	‐0.40%
			38 (Patient 3)				98.90%	98.20%		N/R	4.00%
Maeda et al.^20^	Proton	78 Gy/39 fr. (74 Gy/ 37fr.if low risk)	375	Bone matching	CT‐on‐rail	V95% of CTV > 95% (Rate)	N/R	90.40%	V77% of rectum < 18% (Rate)	N/R	66.10%
				Prostate matching			N/R	98.70%		N/R	86.10%
Rucinski et al.^25^	Scanning CIRT w/ spacer gel	N/R	19 (10 patients)	Bone matching	CT in the next room	V95% of CTV	N/R	99.8 ± 3.2%	V90% of rectum	1.0 ± 1.1 ml	1.1 ± 2.1 ml
	Scanning CIRT w/o spacer gel		50 (9 patients)		CT‐on‐rail		N/R	99.9 ± 2.5%		5.9 ± 2.6 ml	10.2 ± 10.4 ml
Yokoyama et al.^23^	Passive CIRT	34.4 Gy/ 8 fr. (Initial)	118 (10 patients)	Bone matching	CT‐on‐rail	V95% of prostate (Vertical beam)	100 ± 0%	99.89 ± 0.62%	V95% of rectum (Vertical beam)	2.03 ± 0.48 ml	1.93 ± 1.25 ml
						V95% of prostate (Horizontal beam)	100 ± 0%	100.00 ± 0.00%	V95% of rectum (Horizontal beam)	1.93 ± 0.38 ml	1.88 ± 0.96 ml
		17.2 Gy/ 4fr. (Boost)				V95% of prostate (Vertical beam)	99.89 ± 0.07%	95.95 ± 5.81%	V95% of rectum (Vertical beam)	0.01 ± 0.02 ml	0.37 ± 0.69 ml
						V95% of prostate (Horizontal beam)	99.99 ± 0.02%	97.88 ± 3.87%	V95% of rectum (Horizontal beam)	0.03 ± 0.04 ml	0.43 ± 0.65 ml
This study	Scanning CIRT	51.6 Gy/ 12 fr.	131 (11 patients)	Bone matching	CT‐on‐rail	V95% of CTV	100 ± 0%	98.8 ± 3.49%	V80% of rectum	3.91 ± 0.88 ml	4.39 ± 3.96 ml
						V90% of CTV	100 ± 0%	99.5 ± 2.15%			
(Criteria of this study)						V95% of CTV	≧ 95%		V80% of rectum	< 10 ml	
						V90% of CTV	≧ 98%				

Abbreviations: CIRT, carbon‐ion radiotherapy; CT, computed tomography; CTV, clinical target volume; IMRT, intensity‐modulated radiotherapy; N/R, not reported; PTV, planning target volume; SD, standard deviation.

Scanning CIRT is assumed to require higher level of reproducibility than passive CIRT because scanning CIRT can deliver a high dose matching the target shape. In this study, highly reproducible dose distributions were observed regarding both target (prostate and proximal seminal vesicle) coverage and rectal sparing, in line with prior findings. Furthermore, in this study, we applied the bone matching method, and we did not use any fiducial markers which have a low but certain risk of complications,^33^ resulting in less invasive treatment.

Daily variations of the bladder and rectal volumes were assessed as factors affecting CTV and rectal dose reproducibility in this study. The variation of daily bladder volumes was not significantly correlated with the CTV and rectal doses. Concerning the rectal volume, although its daily variation was not correlated with V95% of CTV (R = 0.20), the rate of CTV unacceptance was significantly higher in cases in which the rectal volume was lower than that for planning CT than in cases in which the volume was increased. Additionally, a strong correlation was observed between the reduction of the rectal volume from planning CT and movement of the CTV center to the posterior side. Thus, reduction of the rectal volume leads to the movement of the CTV to the posterior side, thereby reducing the reproducibility of the CTV dose. Conversely, a strong correlation was noted between the increases of the rectal volume and rectal dose. Therefore, although increases of the rectal volume should be avoided because of the resulting increase of the rectal dose, excessive control (reduction) of the rectal volume in the actual treatment session compared with treatment planning should be avoided to prevent the degradation of CTV coverage and a subsequent increase of cancer recurrence. In this study, as countermeasures against rectal gas and stool, patients used probiotics, laxatives (MgO), and antifoaming agents during the treatment period and enema in planning CT and the actual treatment session if defecation did not occur 24 hr. Meanwhile, if the amount of rectal gas or stool was large and unacceptable, defecation or drainage using a Nelaton catheter was performed. Thus, the reproducibility of the CTV and rectal doses was well balanced using the aforementioned approach.

This study had several limitations. We did not consider intrafractional organ motion, which may lead to differences between the moment of actual treatment and in‐room CT images.^34^ Additionally, some biases concerning adherence to the premedication and pretreatment procedure could have influenced the results.

Here, we reported the acceptable robustness of scanning CIRT for prostate cancer using a less invasive marker‐free bone matching method. Because no report has described the robustness of scanning CIRT for prostate cancer using in‐room CT datasets of entire treatment sessions, this study obtained important findings. Based on these results, we have stopped obtaining daily in‐room CT images for prostate cancer patients. Currently, we perform in‐room CT as needed, which has improved the throughput of treatment. Although the robustness of the marker‐free and kV only image guidance of bone matching method was demonstrated in the current treatment schedule of 12 sessions, some unacceptable sessions were observed. Therefore, if we consider the implementation of less fractionated schedules such as ultrahypofraction in the future, improvements in matching techniques are desired.

## 5 CONCLUSION

We demonstrated acceptable interfractional robustness based on the dose distribution in scanning CIRT for prostate cancer using a marker‐free bone matching method. Improvements of the matching techniques aiming for less hypofractionated schedules are desirable.

## AUTHOR CONTRIBUTION

KT collected and analyzed the data and drafted the manuscript. KT, SM, and YK contributed to the study design. KK, WA, YT, NM, IS, DY, KI, YT, and TK collected and analyzed the data. SM aided in writing the manuscript and contributed to the final draft of the manuscript. HK and TO contributed to the final draft of the manuscript. All authors read and approved the final manuscript.

## CONFLICT OF INTEREST

Dr. Hiroyuki Katoh, Dr. Daisaku Yoshida and Dr. Shinichi Minohara receive research funding from Toshiba Energy Systems and Solutions Corporation.

## Data Availability

The data that support the findings of this study are available from the corresponding author upon reasonable request.
